# Correction: Intracellular cGMP increase is not involved in thyroid cancer cell death

**DOI:** 10.1371/journal.pone.0349706

**Published:** 2026-05-20

**Authors:** The PLOS One Staff

## Notice of Republication

This article was republished on May 5, 2026, to correct an error in the first author's name. The correct name is: Sara D’Alessandro. The correct citation is: D’Alessandro S, Paradiso E, Lazzaretti C, Sperduti S, Perri C, Antoniani F, et al. (2023) Intracellular cGMP increase is not involved in thyroid cancer cell death. PLoS ONE 18 (3): e0283888. https://doi.org/10.1371/journal.pone.0283888

The copyright statement for this article has also been updated to include the corrected author’s name.

The publisher apologizes for the error. Please download this article again to view the correct version. The originally published, uncorrected article and the republished, corrected articles are provided here for reference.

## Supporting information

S1 File Originally published, uncorrected article. (PDF)

S2 File Republished, corrected article. (PDF)
