# Supplementary figures and images for: Intracellular cGMP increase is not involved in thyroid cancer cell death
Source: PLoS One. 2023 Mar 30;18(3):e0283888. doi: 10.1371/journal.pone.0283888 (PMC10062617; doi:10.1371/journal.pone.0283888)

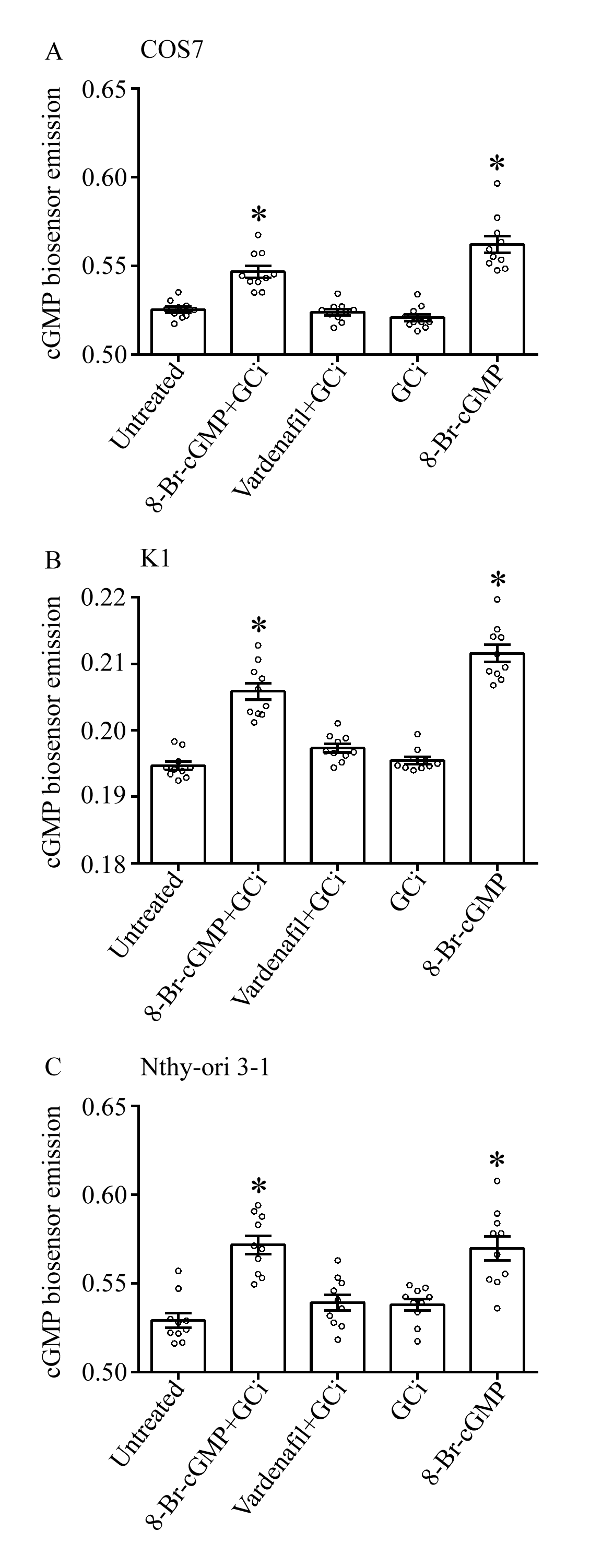

Supplement: S1 Fig — (TIF) [file pone.0283888.s001.tif]

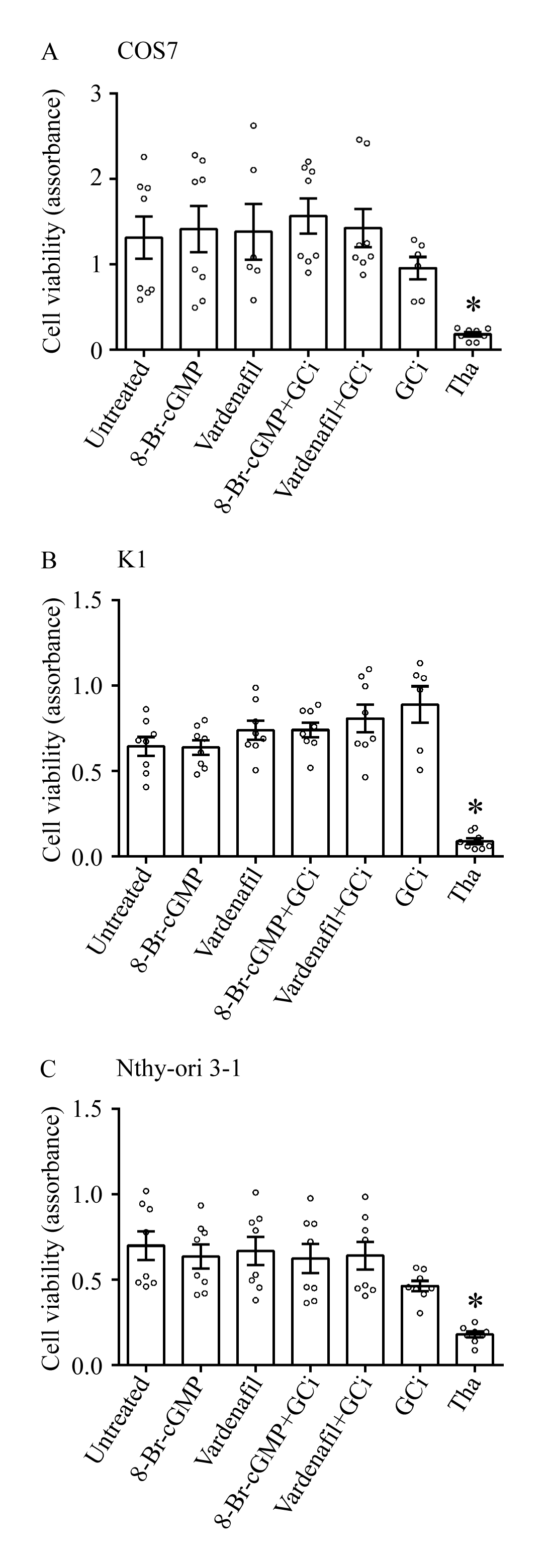

Supplement: S2 Fig — (TIF) [file pone.0283888.s002.tif]

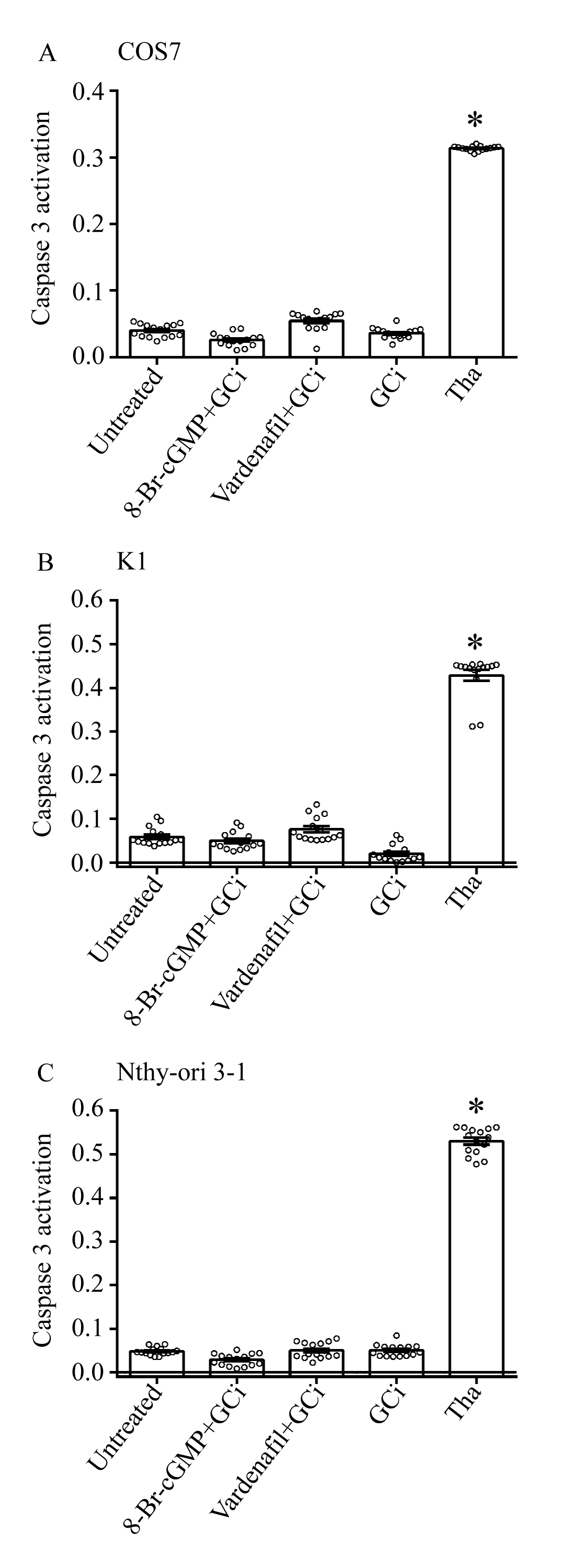

Supplement: S3 Fig — (TIF) [file pone.0283888.s003.tif]

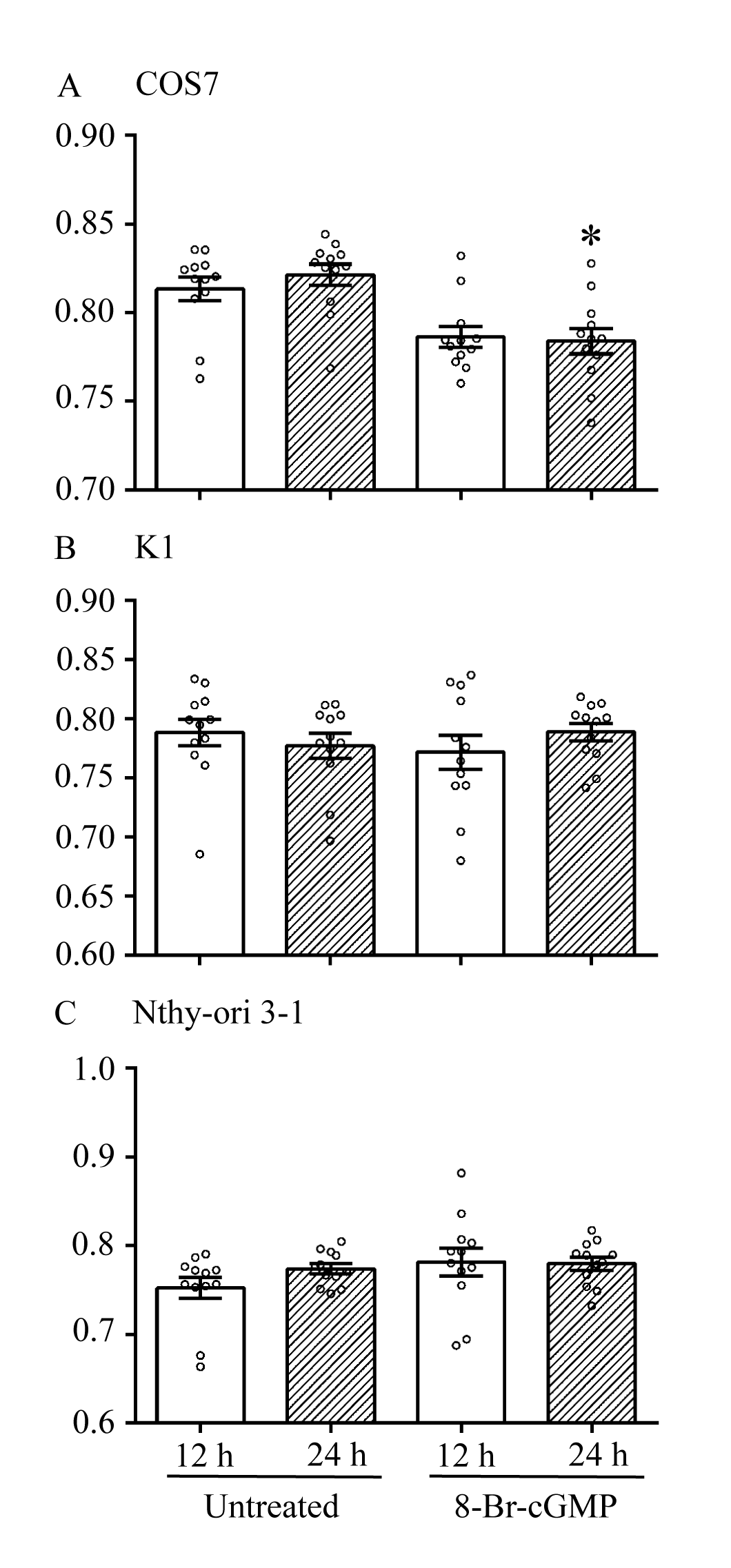

Supplement: S4 Fig — (TIF) [file pone.0283888.s004.tif]

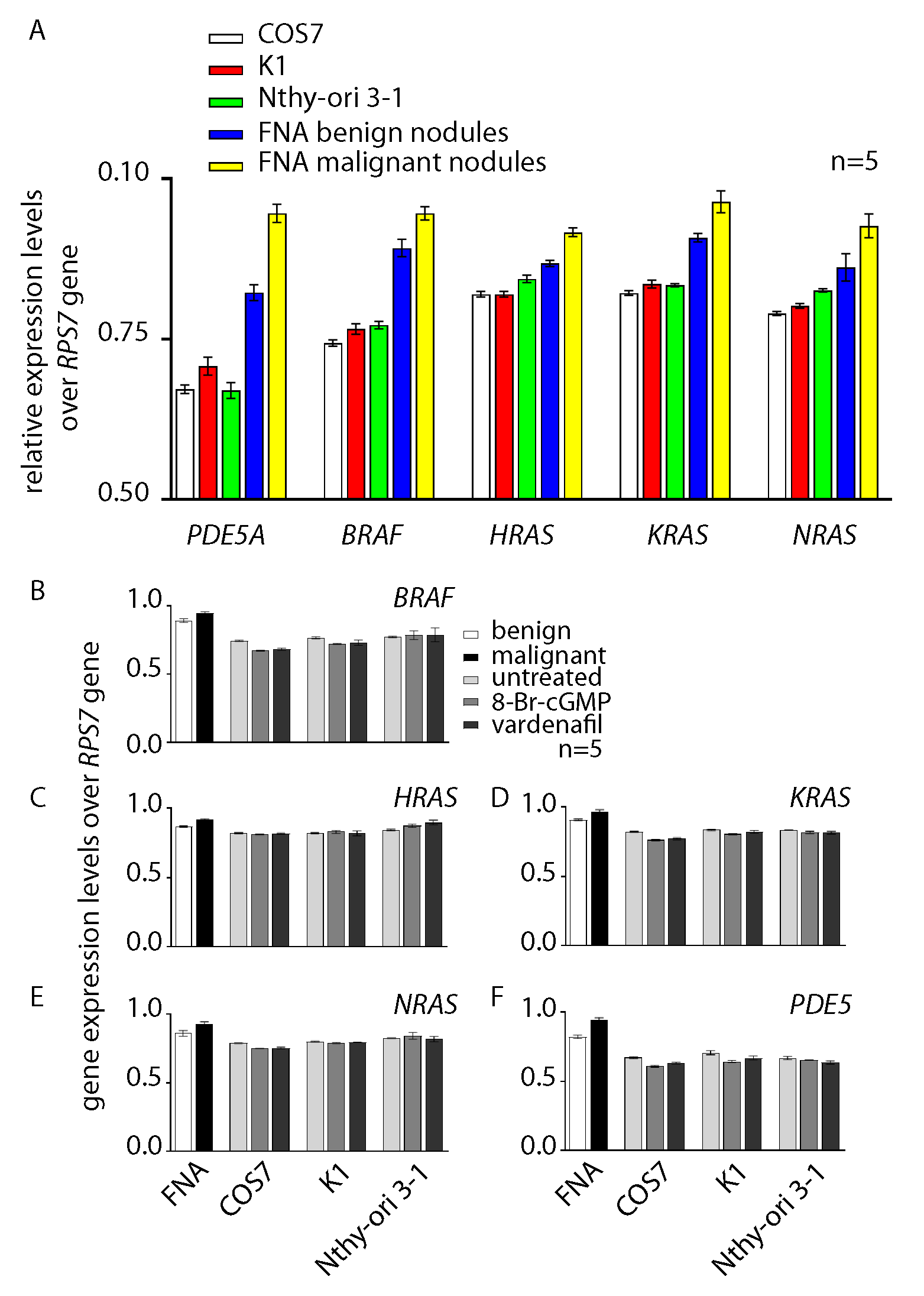

Supplement: S5 Fig — (TIF) [file pone.0283888.s005.tif]
