# Supplementary material for: Intracellular cGMP increase is not involved in thyroid cancer cell death
Source: PLoS One. 2023 Mar 30;18(3):e0283888. doi: 10.1371/journal.pone.0283888 (PMC10062617; doi:10.1371/journal.pone.0283888)
Supplement: S1 File — (DOCX) [file pone.0283888.s006.docx]

**Supplementary materials and methods**

**Gene expression**

To evaluate the effect of increased intracellular cGMP concentration on the expression of *TP53* by RealTime-PCR (RT-PCR), thyroid and COS7 cell lines were seeded in 24-wells plates (5x10^4^ cells/well) and, after over-night starvation, cells were stimulated for 2, 4, 6, 12 and 24 hours with 8-Bromo-cGMP 1 µM. Stimulation were blocked in -80°C after removing the supernatant from each well. RNA was extracted using RiboEx (GeneAll Biotechnology) and following the guidelines provided by the manufacturer. The obtained RNA was then quantified with NanoDrop 2000 (Thermo Fisher Scientific, USA) in order to proceed with the reverse transcription with *High-Capacity cDNA Reverse Trascription Kit* (Applied Biosistem, Thermo Fisher Scientific). Gene expression of *TP53* was evaluated using primers *forward (Fwd)* 5’-GGAGACACCGCTTGGAACTA-3’ and *reverse (Rev)* 5’-AATCTTTGTTCCCGTTCCTCA-3’ while, for the housekeeping *RPS7* gene primers *Fwd* 5’-AATCTTTGTTCCCGTTCCTCA-3’ and *Rev* 5’-CGAGTTGGCTTAGGCAGAA-3’ were used. In a separate experimental stage, *PDE5*, *BRAF*, *KRAS*, *NRAS* and *HRAS* gene expression levels were evaluated as well, in the cell lines and thyroid cells from benign and malignant nodules collected by fine-needle aspiration (FNA). Primer sequences used in this analysis were: *PDE5A* gene *Fwd* 5'-CACTTGCCCAGCTTTACTGC-3' and *Rev* 5'-GGAGAGGCCACTGAGAATCTG-3'; *BRAF* gene *Fwd* 5'-AGGTGATTTTGGTCTAGC-3' and *Rev* 5'-ATCCAGACAACTGTTCAA-3'; *HRAS* gene *Fwd* 5'-AAGCAGGTGGTCATTGATGG-3' and *Rev* 5'-TGATGGCAAACACACACAGG-3'; *KRAS* gene *Fwd* 5'-TTATAAGGCCTGCTGAAAATGACTGAA-3' and *Rev* 5'-TGAATTAGCTGTATCGTCAAGGCACT-3'; *NRAS* gene *Fwd* 5'-CTGGGTTCTTCCACAGCACA-3' and *Rev* 5'-TTCACGTTTGCGGTTTGGTT-3'. *RPS7* gene was used as the housekeeping. RT-PCR was performed on CFX96 Time System (Bio-Rad) thermocycler with the following cycle setting: 45 cycles of 3 seconds each at 95°C for *melting*, 5 seconds for *annealing* cycle and 5 seconds for *extension*, both at 65°C. Data obtained were elaborated by GraphPad Prism 6.0 (GraphPad Prism Software Inc., La Jolla, CA, USA) as ratio between gene of interest Cycle threshold (Ct) average and its housekeeping Ct.

**Ethics Committee approval for primary thyroid cells**

Cells from benign and malignant thyroid nodules were obtained by FNA, from patients of the Unit of Endocrinology, Ospedale di Baggiovara (Modena, Italy), under written consent. The study protocol was approved by the local Ethics Committee of Modena (reference number 122/08).
