# Supplementary material for: Intracellular cGMP increase is not involved in thyroid cancer cell death
Source: PLoS One. 2023 Mar 30;18(3):e0283888. doi: 10.1371/journal.pone.0283888 (PMC10062617; doi:10.1371/journal.pone.0283888)
Supplement: Attachment — Submitted filename: Response to Reviewers.docx [file pone.0283888.s007.docx]

**Response to Reviewers**

PONE-D-22-29667

**cGMP is not involved in thyroid cancer cell death**

Sara D’Alessandro, Elia Paradiso, Clara Lazzaretti, Samantha Sperduti, Carmela Perri, Francesco Antoniani, Sara Righi, Manuela Simoni, Giulia Brigante, Livio Casarini

**Reviewer #1**

It might be a good idea for the follow-up study to repeat the experiments adding PPC-1, BCPAP and 8505c cell lines to make sure the discrepancies you found are not due to some "technical issues". Moreover, using an extended panel of the cell lines could be instrumental in order to focus on the cell-specific nature of the drug effect on tumors

Thanks to this Reviewer for her/his helpful suggestions. A further characterization of our three cell lines has been additionally provided, and results were compared to those from primary thyroid cells obtained by fine-needle aspirations (supplementary figure S5). We feel that these data reliably confirm that we are using samples expressing key genes at levels overall homogeneous with those of primary cells. Thus, these data should be suggestive of overall similar behaviour among our cell lines and thyroid cells.

We agree with the Reviewer that more comprehensive conclusions could be achieved by extending the panel of cell lines. At the same time, we are confident that our selection of three different cell lines provided consistent and converging results supporting our conclusions. The number of available cell lines is huge; we feel to have used a number of cell lines to strengthen our results, although opposite results coud be reasonably expected using different cells and we have discussed this issue.

**Reviewer #2**

In this study, the authors attempted to clarify the role of PDE5i and cGMP in thyroid cancer cell biology in vitro. The story of the paper provides some theoretical basis for the safety of PDE5i treatment for erectile dysfunction patients with thyroid cancer, it is of some clinical value, and considerably simple and easy to be understood. However, due to the simpleness, the story should be more rigorous. As commented below, there are not a few compromised points in this manuscript. Therefore, in my view,this manuscript in its current form, is unsuitable for Journal of PLOS ONE.

1)The title of the paper is not strict. If they only show results using PDE5i inhibitors or cGMP analogues which lead to the elevation of cGMP, “Increased cGMP is not involved in thyroid cancer cell death” may be more suitable. It should be more directly linked to the contents of the paper.

Thanks to the Reviewer for her/his careful observation, the title has been changed accordingly to emphasize the lack of effect related to cGMP increase, which is the main focus of the paper. In any case, please, note that we have used the guanylate cyclase inhibitor (GCi) NS 2028 (supplementary figure S2) to block the cGMP production, leading to no changes of cell viability.

2)The PDE5 activity assay and PDE5 mRNA level should be tested to confirm the effect of PDE5i vardenafil in thyroid cancer cells.

As requested, PDE5 mRNA levels were additionally showed (Supplementary Figure S5) and they are not statistically different from those detected cells collected from benign and malignant thyroid nodules by fine-needle aspiration (FNA).

3) Currently, there are inadequate data to make any conclusive statements “cGMP is not involved in thyroid cancer cell death” by only utilizing the PDE5i and cGMP analog. Specific inhibition (knockdown) of PDF5 using RNA interference should be performed to confirm the role of PDE5 inhibition on thyroid cancer cell.

The conclusive statement as been referred now to the “cGMP increase” througout the paper (abstract, aim of the study, discussion and conclusions sections), rather than cGMP itself.

PDE5 knockdown by siRNA may certainly consist in a further control experiment for our results. However, we respectfully disagree with the Reviewer about the requirement of this experiment. Vardenafil is a very well established, potent and selective pharmacological inhibitor of the PDE5 enzyme, as extensively described by the literature (e.g. *Andersson. PDE5 inhibitors - pharmacology and clinical applications 20 years after sildenafil discovery. Br J Pharmacol. 2018;175(13):2554-2565*). We see no reason to doubt about vardenafil targeting, indeed our BRET data showed that cGMP increases under cell treatment with this inhibitor (Figure 1). These results certify the success of the treatment, and we feel that further approaches to inhibit the action of PDE5 are not of central relevance to support our conclusions, especially in light of similar results obtained upon cell treatment with 8-Br-cGMP.

4) Have the authors investigated expression of PDE5 and cGMP in thyroid cancer tissues or cells compared with normal or other cancer cell lines/tissues that have been reported to be impacted by PDE5i. These types of experiments would significantly enhance the clinical significance of the data. Is there a possibility that the expression level of PDE5 is relatively low or absent in thyroid cancer, thus, further inhibition would unlikely create a greater functional effect. The impact of PDE5 overexpression on thyroid cancer cell biology is required to address.

As requested, we have provided the evaluation of *PDE5* mRNA expression levels, as well as *BRAF* and those of RAS protein-coding genes, which are known to regulate cell growth, proliferation, and migration, and linked to guanosine di/triphosphate metabolism (online supplementary Figure S5). All these genes have overall similar expression levels in primary thyroid cells and in our cell lines. Thanks to the Reviewer for this helpful suggestion.

5) In the supplementary figure 1, one more set using vardenafil should be designed.

The required experiment is already showed in the Figure 1, panels B, D and F, where different vardenafil concentrations were tested.

**Reviewer #3**

D’Alessandro and colleagues present a detailed cellular analysis of cGMP modulation in cell lines including kidney fibroblast, normal thyroid and thyroid cancer. Through thapsigargin control and vardenafil stimulation known to induce cGMP pathway activation in penile, prostatic and cardiac cells, the authors convincingly show that cGMP levels are unchanged by biosensor emission without effects on viability. In complementary studies, the authors show that different from literature reports, PDE5 inhibitor vardenafil did not impact ERK phosphorylation by Western blotting, and weakly impacts caspase 3 activity by as specific biosensor and BRET. The authors acknowledge the limitations of the study in terms of vardenafil use as unique agent with the PDE5 drug class, include appropriate negative and positive controls in each experiment. The title is provocative, but supported by the conclusions and adds to the thyroid cancer literature in particular.

-Discussion of specific areas for improvement

--Major Issues:

The readership would be interested in the effect of cGMP modulation on known targets of differentiated papillary thyroid cancer treatment such as RAS or BRAF. The authors partially address BRAF signaling in their pERK1/2 studies presented in Figure 3. Did the authors explore or reference RAS or BRAF activation or protein expression across their cell line panel? If not, would suggest discussing this as a limitation of the study.

Thanks for this helpful suggestion. We have added the characterization of *KRAS*, *NRAS*, *HRAS* and *BRAF* expression levels and a brief discussion has ben added as well (Supplementary Figure S1).

--Minor Issues

*As a reviewer I appreciate including the number of data points comprising each bar graph. For clarity, I would suggest mentioning the n of biological samples in the figure captions. This is done inconsistently through the figures, for instance mentioned in the caption of Figure 2 but not 1, 3 or 4.

The number of replicates have been added in the captions, thank you.

*The caption of figure 2 reports n=3, but the data points per bar number close to 6 in panels A-D. Can the authors clarify in the captions or methods this discrepancy? Did the authors perform 3 cellular experiments per condition with duplicates?

We have indicated the number of independent exeriments (from 6 to 8) in the figure caption, there was a mistake indeed. Thanks to the Reviewer.

**Journal Requirements:**

When submitting your revision, we need you to address these additional requirements.

1. Please ensure that your manuscript meets PLOS ONE's style requirements, including those for file naming. The PLOS ONE style templates can be found at

https://journals.plos.org/plosone/s/file?id=wjVg/PLOSOne_formatting_sample_main_body.pdf and

https://journals.plos.org/plosone/s/file?id=ba62/PLOSOne_formatting_sample_title_authors_affiliations.pdf

Done.

2. Please clarify from where you obtained the cell lines used in your study.

The origin of cell lines is indicated.

3. We note that the grant information you provided in the ‘Funding Information’ and ‘Financial Disclosure’ sections do not match.

Funding details have been edited accordingly.

When you resubmit, please ensure that you provide the correct grant numbers for the awards you received for your study in the ‘Funding Information’ section.

Done.

4. Thank you for stating the following in the Acknowledgments Section of your manuscript:

“University of Modena and Reggio Emilia, Modena, Italy, and the Italian Ministry of University and Research.”

We note that you have provided additional information within the Acknowledgements Section that is not currently declared in your Funding Statement. Please note that funding information should not appear in the Acknowledgments section or other areas of your manuscript. We will only publish funding information present in the Funding Statement section of the online submission form.

Please remove any funding-related text from the manuscript and let us know how you would like to update your Funding Statement. Currently, your Funding Statement reads as follows:

“Italian Ministry of University and Research supported the Department of Biomedical, Metabolic and Neural Sciences (University of Modena and Reggio Emilia, Italy) in the context of the Departments of Excellence Programme. No specific authors were funded. Grant number is not available and the Funder did not play any role in the study design, data collection and analysis, decision to publish, or preparation of the manuscript.”

Please include your amended statements within your cover letter; we will change the online submission form on your behalf.

Thank you. We have changed the “Acknowledgements” section stating that “Authors are grateful to the Italian Ministry of University and Research”.

Funding information are now available only in the online submission system and are unchanged.

5. In your Data Availability statement, you have not specified where the minimal data set underlying the results described in your manuscript can be found. PLOS defines a study's minimal data set as the underlying data used to reach the conclusions drawn in the manuscript and any additional data required to replicate the reported study findings in their entirety. All PLOS journals require that the minimal data set be made fully available. For more information about our data policy, please see http://journals.plos.org/plosone/s/data-availability.

We have stored source files and raw data in the “Figshare” publicly available online repository (doi: 10.6084/m9.figshare.22151912), and we have indicated this issue in the text.

Upon re-submitting your revised manuscript, please upload your study’s minimal underlying data set as either Supporting Information files or to a stable, public repository and include the relevant URLs, DOIs, or accession numbers within your revised cover letter. For a list of acceptable repositories, please see http://journals.plos.org/plosone/s/data-availability#loc-recommended-repositories. Any potentially identifying patient information must be fully anonymized.

As written above, source files and raw data are now stored in a publicly available online repository.

Important: If there are ethical or legal restrictions to sharing your data publicly, please explain these restrictions in detail. Please see our guidelines for more information on what we consider unacceptable restrictions to publicly sharing data: http://journals.plos.org/plosone/s/data-availability#loc-unacceptable-data-access-restrictions. Note that it is not acceptable for the authors to be the sole named individuals responsible for ensuring data access.

Source files and raw data are now stored in a publicly available online repository.

We will update your Data Availability statement to reflect the information you provide in your cover letter.

Thank you.

6. We note that you have stated that you will provide repository information for your data at acceptance. Should your manuscript be accepted for publication, we will hold it until you provide the relevant accession numbers or DOIs necessary to access your data. If you wish to make changes to your Data Availability statement, please describe these changes in your cover letter and we will update your Data Availability statement to reflect the information you provide.

Source files and raw data are now stored in a publicly available online repository and accession details indicated in the paper.

Changes of Data Availability statement have been described in the cover letter.

7. PLOS ONE now requires that authors provide the original uncropped and unadjusted images underlying all blot or gel results reported in a submission’s figures or Supporting Information files. This policy and the journal’s other requirements for blot/gel reporting and figure preparation are described in detail at https://journals.plos.org/plosone/s/figures#loc-blot-and-gel-reporting-requirements and https://journals.plos.org/plosone/s/figures#loc-preparing-figures-from-image-files. When you submit your revised manuscript, please ensure that your figures adhere fully to these guidelines and provide the original underlying images for all blot or gel data reported in your submission. See the following link for instructions on providing the original image data: https://journals.plos.org/plosone/s/figures#loc-original-images-for-blots-and-gels.

Uncropped and other original images are provided in the publicly available online repository now.

In your cover letter, please note whether your blot/gel image data are in Supporting Information or posted at a public data repository, provide the repository URL if relevant, and provide specific details as to which raw blot/gel images, if any, are not available. Email us at plosone@plos.org if you have any questions.

Done.
