# Supplementary material for: Correction: Intracellular cGMP increase is not involved in thyroid cancer cell death
Source: PLoS One. 2026 May 20;21(5):e0349706. doi: 10.1371/journal.pone.0349706 (PMC13189302; doi:10.1371/journal.pone.0349706)
Supplement: S1 File — (PDF) [file pone.0349706.s001.pdf]

PLOS ONE

RESEARCH ARTICLE

Intracellular cGMP increase is not involved in thyroid cancer cell death

Sara D' Alessandro[1,2], Elia Paradiso[1], Clara Lazzaretti[1], Samantha Sperduti[1,3], Carmela Perri[1], Francesco Antoniani[1], Sara Righi[1], Manuela Simoni[1,3,4], Giulia Brigante[1,4], Livio Casarini[1,3]*

1 Unit of Endocrinology, Department of Biomedical, Metabolic and Neural Sciences, University of Modena and Reggio Emilia, Modena, Italy, **2** International PhD School in Clinical and Experimental Medicine (CEM), University of Modena and Reggio Emilia, Modena, Italy, **3** Center for Genomic Research, University of Modena and Reggio Emilia, Modena, Italy, **4** Unit of Endocrinology, Department of Medical Specialties, Azienda Ospedaliero-Universitaria di Modena, Modena, Italy

* livio.casarini@unimore.it

Check for updates

OPEN ACCESS

Citation: Alessandro SD, Paradiso E, Lazzaretti C, Sperduti S, Perri C, Antoniani F, et al. (2023) Intracellular cGMP increase is not involved in thyroid cancer cell death. PLoS ONE 18(3): e0283888. https://doi.org/10.1371/journal.pone.0283888

Editor: Avaniyapuram Kannan Murugan, King Faisal Specialist Hospital and Research Center, SAUDI ARABIA

Received: October 27, 2022

Accepted: March 20, 2023

Published: March 30, 2023

Peer Review History: PLOS recognizes the benefits of transparency in the peer review process; therefore, we enable the publication of all of the content of peer review and author responses alongside final, published articles. The editorial history of this article is available here: https://doi.org/10.1371/journal.pone.0283888

Copyright: © 2023 Alessandro et al. This is an open access article distributed under the terms of the Creative Commons Attribution License, which permits unrestricted use, distribution, and reproduction in any medium, provided the original author and source are credited.

Data Availability Statement: All source files and raw data are available from the Figshare database (doi: 10.6084/m9.figshare.22151912).

Abstract

Introduction

Type 5 phosphodiesterase (PDE5) inhibitors (PDE5i) lead to intracellular cyclic-guanosine monophosphate (cGMP) increase and are used for clinical treatment of erectile dysfunction. Studies found that cGMP may up/downregulate the growth of certain endocrine tumor cells, suggesting that PDE5i could impact cancer risk.

Aim

We evaluated if PDE5i may modulate thyroid cancer cell growth *in vitro*.

Materials and methods

We used malignant (K1) and benign (Nthy-ori 3–1) thyroid cell lines, as well as the COS7 cells as a reference model. Cells were treated 0–24 h with the PDE5i vardenafil or the cGMP analog 8-br-cGMP (nM-μM range). cGMP levels and caspase 3 cleavage were evaluated by BRET, in cGMP or caspase 3 biosensor-expressing cells. Phosphorylation of the proliferation-associated extracellularly-regulated kinases 1 and 2 (ERK1/2) was evaluated by Western blotting, while nuclear fragmentation by DAPI staining. Cell viability was investigated using 3-(4,5-dimethylthiazol-2-yl)-2,5-diphenyltetrazolium bromide (MTT) assay.

Results

Both vardenafil and 8-br-cGMP effectively induced dose-dependent cGMP BRET signals ($p \leq 0.05$) in all the cell lines. However, no differences in caspase 3 activation occurred comparing PDE5i-treated *vs* untreated cells, at all concentrations and time-points tested ($p > 0.05$). These results match those obtained upon cell treatment with 8-br-cGMP, which failed in inducing caspase 3 cleavage in all the cell lines ($p > 0.05$). Moreover, they reflect the lack of nuclear fragmentation. Interestingly, the modulation of intracellular cGMP levels with

Funding: Italian Ministry of University and Research supported the Department of Biomedical, Metabolic and Neural Sciences (University of Modena and Reggio Emilia, Italy) in the context of the Departments of Excellence Programme. No specific authors were funded. Grant number is not available and the Funder did not play any role in the study design, data collection and analysis, decision to publish, or preparation of the manuscript.

Competing interests: The authors have declared that no competing interests exist.

vardenafil or the analog did not impact cell viability of both malignant and benign thyroid tumor cell lines, nor the phosphorylation of ERK1/2 (p>0.05).

Conclusions

This study demonstrates that increased cGMP levels are not linked to cell viability or death in K1 and Nthy-ori 3–1 cell lines, suggesting that PDE5i do not impact the growth of thyroid cancer cells. Since different results were previously published, further investigations are recommended to clarify the impact of PDE5i on thyroid cancer cells.

Introduction

The 3',5'-cyclic guanosine monophosphate (cGMP) is a second messenger activating the nitric oxide (NO)-dependent soluble guanylyl cyclase (sGC) or the natriuretic peptides (NPs)-dependent particulate guanylyl cyclase (pGC). They have a cGMP-elevating effect, activating second messenger-dependent protein kinases G (PKG), phosphodiesterases (PDEs) and ion channels. Dysregulation of these intracellular signaling pathways may lead to pathological effects, since cGMP is involved in cell proliferation, as well as in cancer development and progression [1]. Its ability to modulate cell proliferation and apoptosis depends on the cell or tumor type, which differently responds to up/downregulation of NO/cGMP pathway [1–3]. For this reason, it is hard to predict how drugs interfering with cGMP cascade, such as PDE5 inhibitors (PDE5i), could impact cancer cell proliferation.

PDE5i were first introduced for the treatment of erectile dysfunction (ED). Vardenafil is one of these synthetic and PDE5-specific compounds that competitively inhibits the hydrolysis of cGMP, thus, increasing the intracellular levels of the second messenger [4]. To date, these kind of drugs are clinically useful for the treatment of pulmonary hypertension, cardiovascular disease, and of benign prostatic hyperplasia (BHP) symptoms [3, 5]. PDE5i are also used to enhance effects of chemotherapeutic medications. In fact, certain tumor cells develop resistance to these drugs over time, due to a molecular mechanism based on ATP-binding cassette (ABC) transponders overexpression [6]. It results in the drug exporting out from the cell, lowering the therapeutic efficacy. In this case, PDE5i acts as a non-toxic transporter modulator and enhancer of drug efficacy [3].

Several studies characterized the cancer cell-specific effect of cGMP pathway, which is linked to proliferation or death. For instance, increasing cGMP levels may upregulate apoptotic events and inhibit tumorigenesis of colon cancer cells [7–10], breast cancer cells [8, 11, 12], gastric cancer cells [8, 13, 14], pancreatic adenocarcinoma cells [15] leukemia and myeloma [3], prostate carcinoma [16] and glioma cells [8]. Moreover, cGMP/PKGII signaling was found to exert inhibitory effects on growth and metastasis in various human tumor cell lines *in vitro*, including lung, hepatic, renal, colon cancer and glioma cell lines [8]. On the contrary, anti-apoptotic effects or increased risk of tumor development were found respectively in ovary carcinoma cells [17] and melanoma [3].

The link between intracellular cGMP levels and thyroid disorders was hypothesized several decades ago [18–20]. This issue was investigated in thyroid nodules of animal models, where the second messenger could modulate the production of inflammatory mediators [21]. Most importantly, studies in human biopsies revealed that increased cGMP levels may trigger autophagic signals counteracting proliferation of papillary thyroid cancer (PTC) cells and preventing their expansion to other tissues and organs [22]. Similar conclusions were achieved by

bioinformatic approaches, suggesting that downregulation of cGMP pathway-related genes may be associated with PTC [23]. However, data about the role of cGMP in PTC, as well as if PDE5i may modulate thyroid tumor cell growth are relatively scarce.

In this *in vitro* study, we explored whether intracellular cGMP increase may be a target for PTC therapy. Intracellular levels of the second messenger were intentionally perturbed by adding a cGMP analog or a PDE5i to cell media, and pro/anti-apoptotic signals were evaluated. To this purpose, the human PTC cell line K1 was used as a model of thyroid malignant cancer, while the follicular epithelial cell line Nthy-ori 3–1 as a benign cell of thyroid origin.

Materials and methods

Source files and raw data are available in an online repository (doi: 10.6084/m9.figshare.22151912).

Reagents

8-br-labeled GMP (8-Br-cGMP) (CAS.N.:51116-01-9, Santa Cruz Biotechnology, Santa Cruz, CA, USA) and vardenafil hydrochloride CRS (CAS.N.: 330808-88-3, Merck KGaA) were used to treat cells (nM-μM concentration range) and induce the increase of intracellular cGMP levels. 4 μM thapsigargin (Tocris Bioscience, Bristol, UK) was used as a positive control in caspase 3 activation analysis, nuclear fragmentation and 3-(4,5-dimethylthiazol-2-yl)-2,5-diphenyltetrazolium bromide (MTT) assay. 0.1 μM phorbol myristate acetate (PMA) (Sigma-Aldrich) and 10 μM of the mitogen-activated protein kinase kinase (MEK) inhibitor U0126 (Sigma-Aldrich) were used as positive and negative controls for ERK1/2 phosphorylation, respectively [24]. 10 μM NS 2028 (CAS.N.: 204326-43-2, Merck KGaA) was used as inhibitor of sGC.

Cultured cell lines

The K1 cell line was used as a model of malignant thyroid tumor, i.e. PTC [25]. The Nthy-ori 3–1 cell line were from immortalized epithelial thyroid cells [26] and served as a model of healthy thyroid. The COS7 cell line was used as a reference cell line and were available in-house. K1 cells were cultured with Dulbecco's modified eagle medium (DMEM), Ham's F12 and MCDB 105 (all from Sigma-Aldrich, a division of Merck KGaA, Darmstadt, Germany) in a 2:1:1 ratio. Nthy-ori 3–1 cells were grown in RPMI-1640, while COS7 were cultured with DMEM (Sigma-Aldrich). All these cell media were enriched by 10% FBS, 2 mM L-glutamine, 100 U/ml penicillin and 50 μg/ml streptomycin (all from Sigma-Aldrich).

Plasmids and transfection

The C163A-pcw107-V5 Caspase 3 BRET biosensor [27] was obtained by Addgene (Watertown, MA, USA) and validated in the present study. GFP^2-GAFa-Rluc sensor was previously described [28] and validated [29]. Transient transfections of cell lines were performed in a 96-well plate using Metafectene PRO (Biontex Laboratories GmbH, Munich, Germany), following the manufacturer's protocol. To this purpose, 50 ng and 200 ng of plasmid coding caspase 3 and cGMP biosensors, respectively, were added to each well after to be mixed with 0.5 μl of Metafectene PRO diluted in FBS-free medium. A volume of 50 μl plasmid-Metafectene PRO mix was added to each well, containing 15.0×10^3 cells, achieving a total volume of 250 μl/well. Cells were incubated 2 days and starved overnight with FBS-free medium before treatments with 8-Br-cGMP and vardenafil.

BRET measurements

Caspase 3 cleavage was evaluated in COS7, K1 and Nthy-ori 3–1 cell lines transiently expressing the specific (C163A)-pcw107-V5 BRET biosensor. In this molecule, a luciferase NanoLuc (NLuc) is fused to the fluorescent acceptor protein mNeonGreen (mNG) via a caspase-cleavable 17 amino acid flexible linker. In the presence of caspase 3, the linker undergoes cleavage, leading to dissociation between NLuc and mNG and resulting in the decrease of BRET signal. cGMP intracellular increase was measured in cell lines transiently expressing the GFP^2-GAFa-Rluc biosensor [29, 30]. Binding between cGMP and the specific GAF domain results in biosensor conformational changes, leading to BRET signal increase. Reactions occurred in the presence of the luciferase enzyme substrate coelenterazine H (NanoLight Technologies, a division of Prolume Ltd., Lakeside, AZ, USA). The cGMP and caspase 3 BRET biosensors detection range was evaluated by dose-response experiments using 8-Br-cGMP and thapsigargin serial dilutions, respectively, after 2 and 4 hours of treatment. Cells were also treated with 8-br-cGMP 1 μM and Vardenafil 50 μM in the presence/absence of Guanylyl cyclase inhibitor 10 μM. 8-br-cGMP 20 μM and Thapsigargin 4 μM were respectively used as positive control for cGMP and caspase3 study to validate the detection range of the sensor. The analysis of cell death signals was extended to pro-apoptotic gene expression (S1 File).

pERK1/2 protein analysis

Proliferative signals were investigated using ERK1/2 phosphorylation, as previously described [31, 32]. To these purposes, cell lines were seeded in 24-well plates ($1.0x10^5$ cells/well) and treated 15 min with 1 μM 8-Br-cGMP or 50 μM vardenafil. Where needed, cells were pretreated 1 h with the NS 2028 GC inhibitor, PMA or U0126 [24]. Cells were lysed in ice-cold RIPA buffer containing protease and phosphatase inhibitors for protein extractions. The phosphorylation of ERK1/2 was evaluated using an anti-pERK1/2 primary antibody (#9101, Cell Signaling Technology Inc., Danvers, MA, USA). Lysed samples were loaded to separate proteins in a 10% sodium dodecyl sulfate (SDS)-polyacrylamide gel electrophoresis and transferred to a polyvinylidene fluoride membrane. The anti-total ERK antibody (#9102, Cell Signaling Technology Inc.) was used as a loading control. Then, membranes were incubated with a secondary anti-rabbit horseradish peroxidase (HRP)-conjugated antibody (#NA9340V; GE HealthCare).

Western blotting signals were revealed upon incubation with ECL chemiluminescent compound (GE HealthCare, Chicago, IL, USA). Images were acquired by the VersaDoc™ MP 4000 System and QuantityOne analysis software (Bio-Rad Laboratories Inc., Hercules, CA, USA).

Cell viability assay

Cell viability was investigated by MTT assay [33], using a known procedure [31]. Cells were seeded in 96-wells plates ($1.0x10^4$ cells/well) and serum-starved over-night before 24-h treatments with increasing concentrations of 8-Br-cGMP (100 nM-50 μM) and vardenafil (100 nM-500 μM). 100 μl/well of assay solution (#M5655; Sigma-Aldrich) was prepared by dissolving 1 mg of MTT powder into 1 ml of cell medium. After 2-h incubation at 37˚C, in the presence of 5% of CO_2, the solution was removed and 100 μl/well of isopropanol was added to dissolve the formazan crystals produced by metabolically active cells. Absorbance was detected at the wavelength of 570 nm by a Victor3 plate reader (Perkin Elmer Inc., Waltham, MA, USA).

Nuclear fragmentation

To investigate whether the intracellular cGMP increase impacts thyroid cancer cell death, $1.5x10^4$ cells/well were seeded in 24-well plates and treated 4–24 h with 1 μM 8-Br-cGMP or 50 μM vardenafil. Thapsigargin served as a positive control inducing nuclear fragmentation [34, 35]. Cells were fixed by 10% formalin and DAPI-stained 20 min. Images were captured by a fluorescence microscope (Nikon Eclipse Ts2R; Nikon Corporation, Tokyo, Japan).

Statistical analysis

Statistical analysis and graphic representation were obtained by GraphPad Prism 9.0 (GraphPad Prism Software Inc., La Jolla, CA, USA) Results were analyzed by Kruskal Wallis test and Dunn's post-test, after to have applied the D'Agostino and Pearson normality test. Differences were considered significant for $p<0.05$.

Results

Intracellular cGMP dose-finding

The optimal 8-Br-cGMP and vardenafil concentration to be used *in vitro* was evaluated by dose-finding experiments in transfected COS7, K1 and Nthy-ori 3–1 cell lines expressing the cGMP BRET biosensor. Cells were treated 2 h with increasing 8-Br-cGMP and vardenafil concentrations before signal detection by BRET. In COS7 and K1 cell lines, the lowest 8-Br-cGMP concentration resulting in detectable signals was 1 μM, while it was 50 μM for vardenafil (Kruskal Wallis test and Dunn's post-test; $p<0.05$). Similar effects were found with 500 μM 8-Br-cGMP in the Nthy-ori 3–1 cell line ($p<0.05$), while vardenafil failed in inducing detectable cGMP increase in this model (Fig 1). cGMP detection by the BRET biosensor was further investigated under cell co-treatment with 10 μM of the sGC inhibitor NS 2028 and 8-Br-cGMP/vardenafil (S1 Fig), confirming the lack of signal in the absence of cGMP.

These experiments served to identify the optimal PDE5i and cGMP analog concentration range to be used in further experiments, where we evaluated if the modulation of intracellular cGMP levels impacts cell viability and death *in vitro*. To this purpose, the 100 nM– 500 μM 8-Br-cGMP/vardenafil range was chosen as the most indicative of cGMP increase. The effective, 1 mM concentration was excluded because it was unnecessarily high to obtain intracellular cGMP increase and because it was near to the vardenafil solubility.

Intracellular increase of cGMP does not affect cell viability

The viability of COS7, K1 and Nthy-ori 3–1 cells was evaluated under 8-Br-cGMP and vardenafil treatment. To this purpose, cells were treated 24 h with the PDE5i or the analog and viability was evaluated by MTT assay. Both the compounds did not reduce cell viability at all the concentration tested (Fig 2), except 500 μM vardenafil (Kruskal Wallis test and Dunn's post-test; $p<0.05$). In this case, results could be biased by sub-optimal dissolution of the compound in the vehicle, which resulted in an opaque solution since the concentration used dropped around the solubility of compound in water [36]. Control experiments confirming that cGMP perturbation does not impact cell viability were performed under sGC blockade by NS 2028 (S2 Fig).

Analysis of the cell viability was deepened by evaluating ERK1/2 phosphorylation, as a hallmark of cell survival [37]. To this purpose, cells treated with 8-Bromo-cGMP and vardenafil were used for Western blotting analysis and pictures were semi-quantified to be represented in graphs (Fig 3). Cells treated with PMA and the MEK inhibitor U0126 served as positive and negative controls, respectively. We found that both 8-Bromo-cGMP and vardenafil failed in

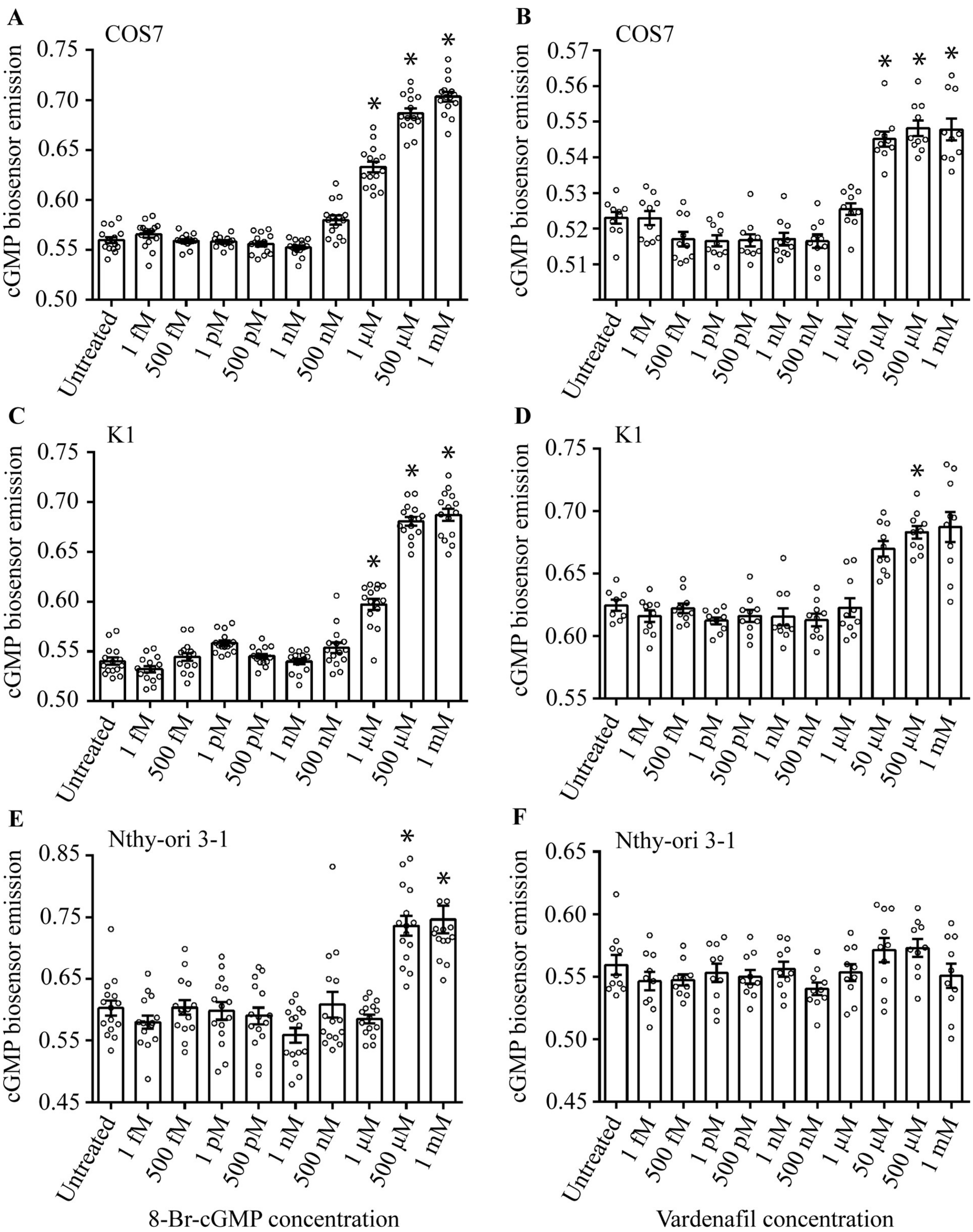

Fig 1. Analysis of intracellular cGMP levels. Transfected cells expressing the cGMP/Rluc-tagged biosensor were treated 2 h with increasing 8-Br-cGMP and vardenafil (fM-mM range). BRET signals indicating intracellular cGMP levels were evaluated and plotted against the concentration of inhibitor/analog. A, B) Data from the COS7 cell line treated with 8-Br-cGMP and vardenafil, respectively; C, D) 8-Br-cGMP- and vardenafil-treated K1 cell line; E, F) Nthy-ori 3–1 cells treated with 8-Br-cGMP and vardenafil. Results were compared by Kruskal Wallis test and Dunn's post-test *versus* untreated samples and statistically significant differences were marked by * ($p<0.05$; means ± SEM; n = 8 to 15).

https://doi.org/10.1371/journal.pone.0283888.g001

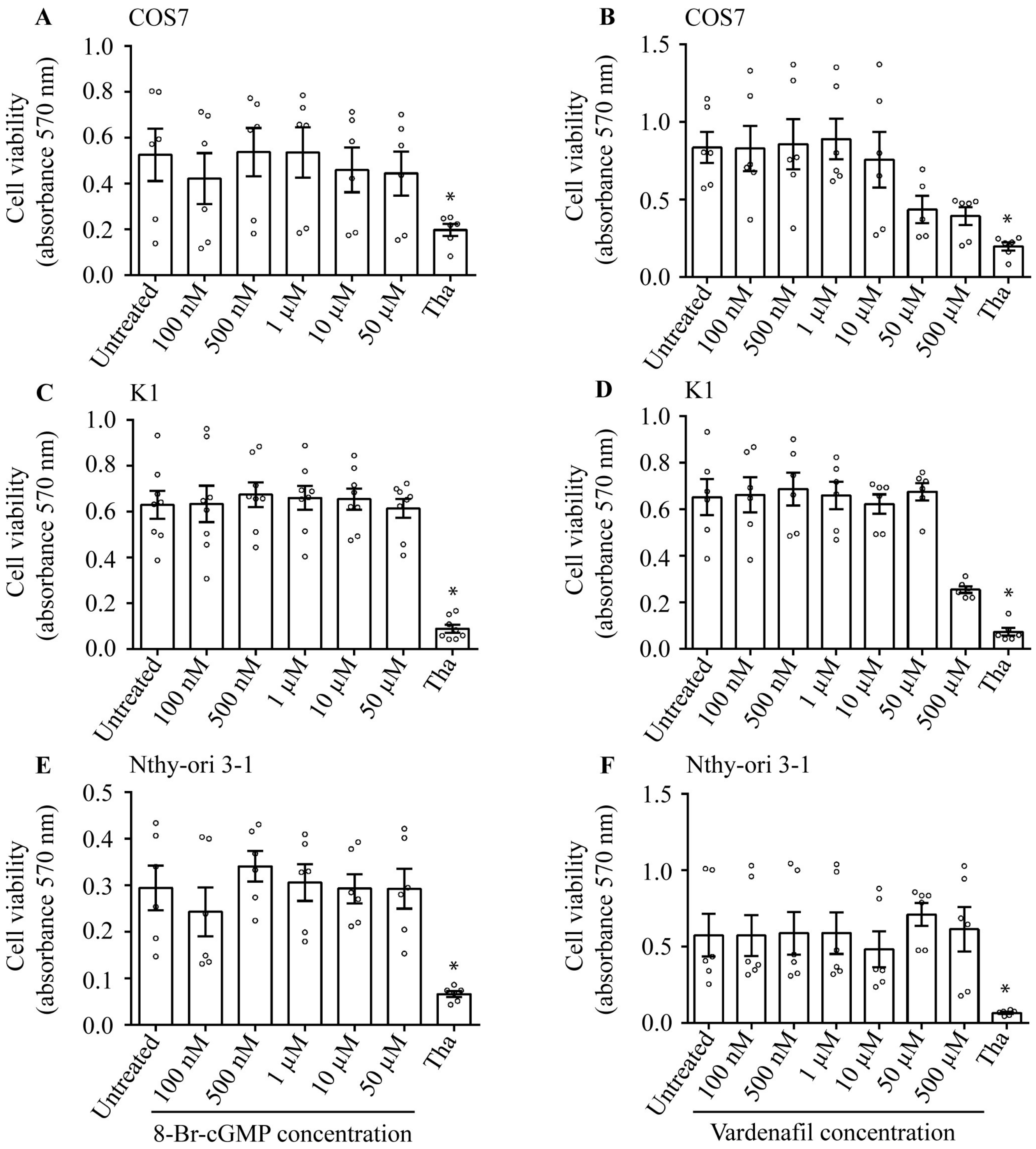

Fig 2. Modulation of cell viability by 8-Br-cGMP/vardenafil. COS7, K1 and Nthy-ori 3–1 cell lines were treated 24 h with increasing 8-Br-cGMP and vardenafil (100 nM– 500 μM range). Cell viability was evaluated by MTT assay and indicated as an absorbance detected at the 570 nm wavelength. 4 μM thapsigargin served as a positive control (Tha) for cell viability decrease and results were compared by Kruskal Wallis test and Dunn's post-test (*significantly different *versus* untreated sample; $p < 0.05$; means ± SEM; n = 6 to 8). A, B) Data from the COS7 cell line treated with 8-Br-cGMP and vardenafil, respectively; C, D) Results from treatments of K1 cells; E, F) Nthy-ori 3–1 cell line.

https://doi.org/10.1371/journal.pone.0283888.g002

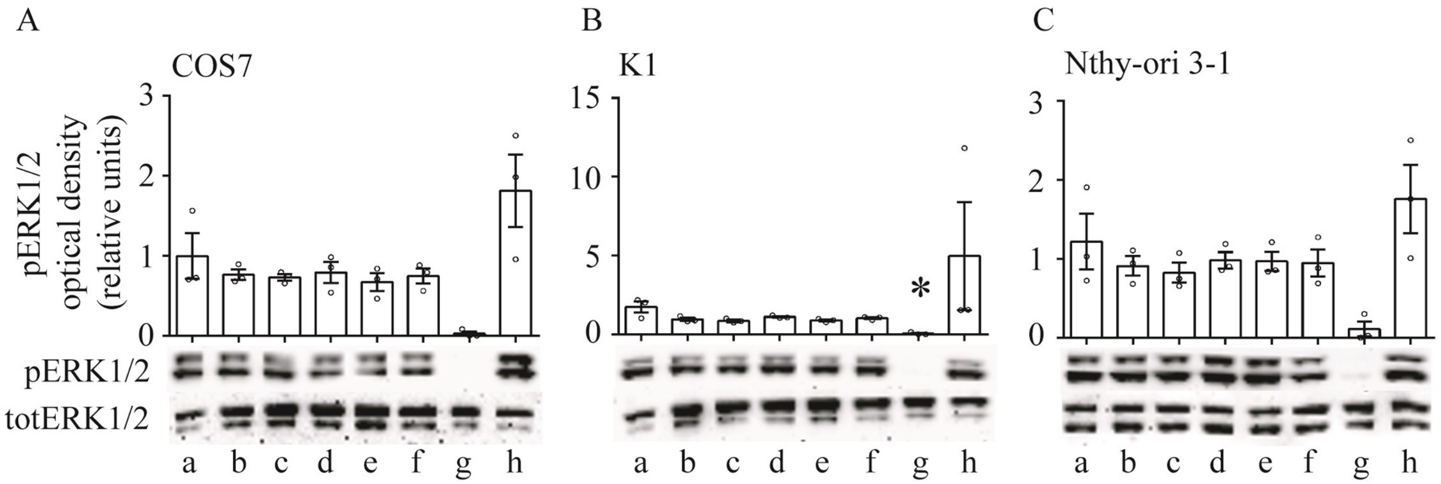

Fig 3. 15-min, 8-Br-cGMP/vardenafil-induced ERK1/2 phosphorylation in the three cell lines. Cells were treated as follow: a) untreated; b) 10 μM 8-Br-cGMP; c) 50 μM vardenafil; d) preincubation with NS 2028 (GCi) and treatment with 8-Br-cGMP; e) preincubation with NS 2028 (GCi) and treatment with vardenafil; f) NS 2028 (GCi); g) MEK inhibitor U0126 was used to deplete ERK1/2 phosphorylation h) PMA was used as positive control of pERK1/2. Data from densitometric analysis were compared by Kruskal Wallis test and Dunn's post-test (*significantly different *versus* untreated samples; $p \geq 0.05$; means ± SEM were superimposed to data points; n = 3).

https://doi.org/10.1371/journal.pone.0283888.g003

modulating pERK1/2 activation compared to the basal level detected in untreated samples (Kruskal Wallis test and Dunn's post-test; $p \geq 0.05$).

8-Bromo-cGMP and vardenafil weakly impact caspase 3 activity

Caspase 3 activation was evaluated in cell lines treated 4 h with the cGMP analog and PDE5i, by BRET. To this purpose, the expression of a specific biosensor for the cleaved caspase 3 activity was induced upon vector transfection. Thapsigargin was used as a positive control [38, 39] We found that both 8-Br-cGMP and vardenafil slightly reduced the caspase 3 activity in COS7 cells, while the activity of the enzyme was potently enhanced upon thapsigargin treatment as in all the other tested cell lines. However, in K1 cells, almost all the concentrations tested of both the cGMP analog and PDE5i induced only a weak increase of the caspase 3 activity, detected as a significantly higher BRET signal (Fig 4; Kruskal Wallis test and Dunn's post-test; $p < 0.05$). Similar results were obtained in Nthy-ori 3–1 cells treated with 100 nM– 1 μM 8-Br-cGMP ($p < 0.05$), while vardenafil induced no effects on caspase 3 activity. The overall weak effect of cGMP perturbation on caspase 3 activity, if any, was confirmed by control experiments using the sGC inhibitor (S3 Fig), and further confirmed by the lack of pro-apoptotic *TP53* gene expression (S4 Fig).

Analysis of caspase 3 activity was followed by evaluations of nuclear damage in cell lines treated 24 h with 1 μM 8-Br-cGMP and 50 μM vardenafil. Cell nuclei were stained by DAPI and pictures captured by a fluorescent microscope. Consistently with the weak or no activation of caspase 3 activity, both treatments with cGMP analog and PDE5i did not induce nuclear fragmentation (Fig 5), and oppositely to what obtained upon cell incubation with thapsigargin (positive control).

Discussion

This study demonstrates that the perturbation of intracellular cGMP levels did not affect pro/anti-apoptotic signals in two *in vitro* models representative of malignant and benign thyroid cells, as well as in the COS7 reference cell line. Both the second messenger analog 8-Br-cGMP and the PDE5i vardenafil failed to induce cell death signals, e.g. consistent caspase 3 activity

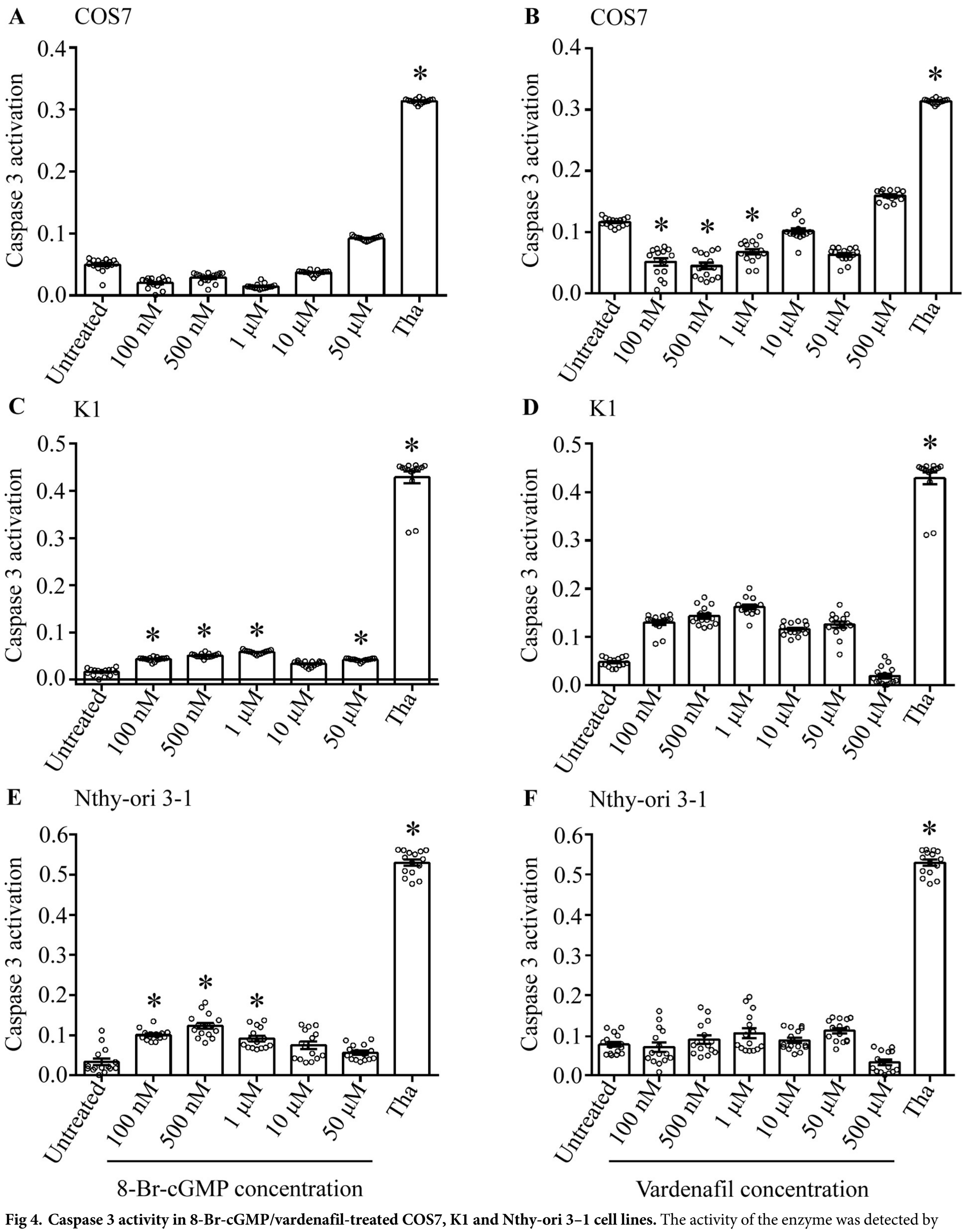

Fig 4. Caspase 3 activity in 8-Br-cGMP/vardenafil-treated COS7, K1 and Nthy-ori 3–1 cell lines. The activity of the enzyme was detected by BRET, in cells treated 4 h with increasing 8-Br-cGMP and vardenafil concentrations (100 nM– 500 μM range) and expressing a specific biosensor. 4 μM thapsigargin served as a positive control (Tha). Results were compared by Kruskal Wallis test and Dunn's post-test (*significantly different *versus* untreated sample; $p < 0.05$; means ± SEM; n = 15). A, B) 8-Br-cGMP- and vardenafil-treated COS7 cells, respectively; C, D) Results from K1 cells; E, F) Nthy-ori 3–1 cell line.

https://doi.org/10.1371/journal.pone.0283888.g004

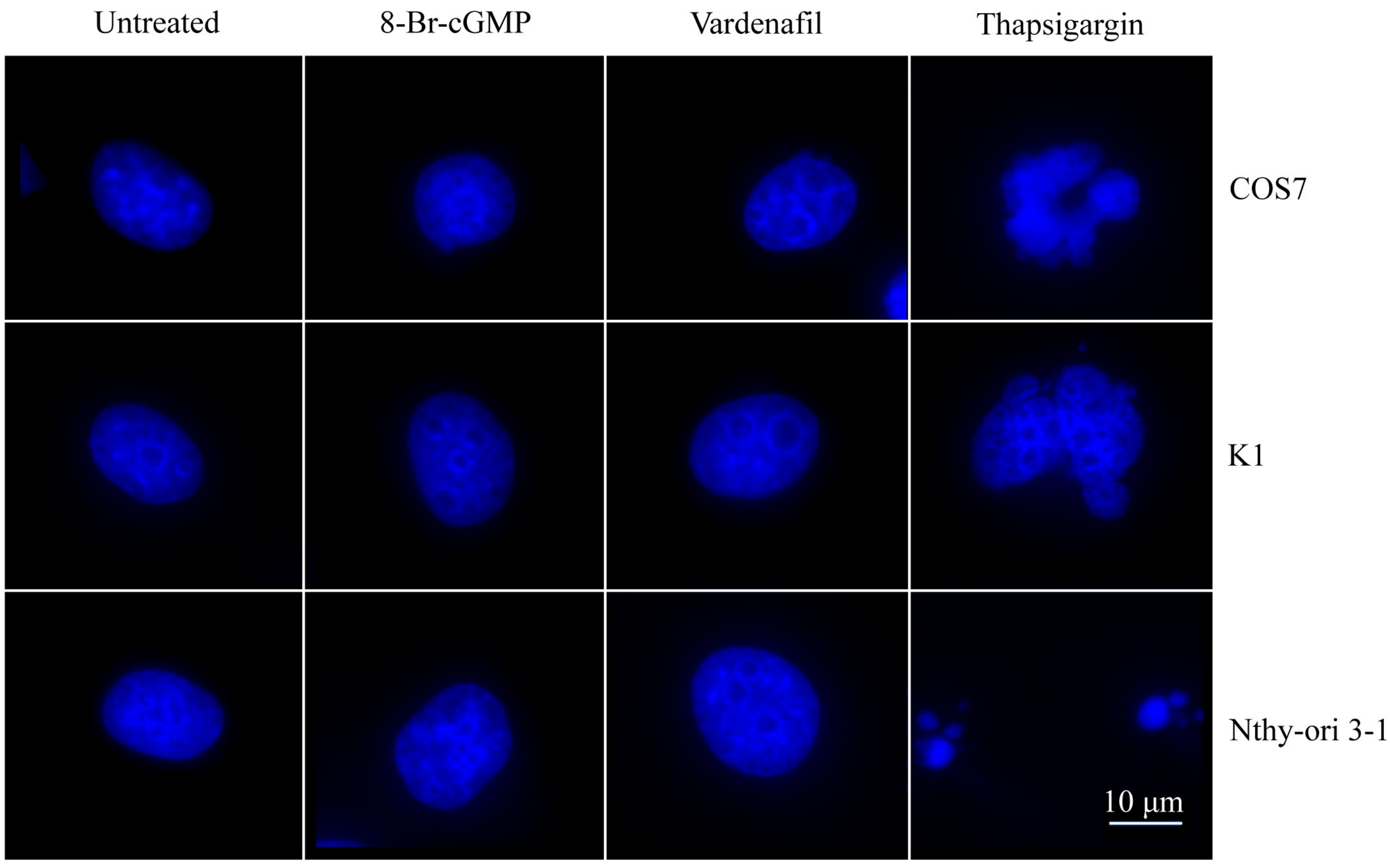

Fig 5. Evaluation of nuclear damage. COS7, K1 and Nthy-ori 3–1 cells were treated 24 h with 1 μM 8-Br-cGMP and 50 μM vardenafil before to be fixed and DAPI stained. 4 μM thapsigargin-treated cells served as positive controls. 60x magnification; scale bar = 10 μM; n = 1.

https://doi.org/10.1371/journal.pone.0283888.g005

and nuclear damage. These data match the failure of sGC inhibitor in enhancing/inhibiting cell viability, confirming a weak effect of cGMP, if any, in the modulation of thyroid cell line metabolism. On one hand, our results suggest that cGMP could not be a target for PTC therapy. On the other hand, they indicate that PDE5i would not impact the growth of malignant thyroid cells and might be used for clinical treatment of erectile dysfunction in PTC patients.

The relevance on this study is found in the increasing use of PDE5i [40], as well as in the dual role of cGMP in tumor cells, where the second messenger may upregulate growth and proliferation or, on the contrary, exert pro-apoptotic effects, depending on the kind of tumor [3, 41]. For instance, cGMP suppresses apoptosis and upregulates proliferative signals in several cancer cells, such as ovarian [17], breast [42], renal carcinoma [43] and head/neck squamous cell carcinoma [44]. For this reason, the use of PDE5i could not be recommended in certain cancer patients. An *in vitro* study demonstrated that the PDE5 inhibition by sildenafil increases the intracellular levels of cGMP which, in turn, potentiates the mitogen-activated protein kinase (MAPK)-dependent melanoma cell growth [45] and increases the risk of tumorigenic effect [46]. However, opposite results were found by studies in different cancer cells and cell lines. cGMP induced apoptosis *via* PKG in certain breast cancer cell lines [11], in colon [47, 48] and gastric cancer cells [49]. Efforts were made to determine why some cancer cells are responsive to cGMP-induced apoptosis. A study suggested that various types of cancer may be selectively sensitive to cGMP-mediated apoptosis, due to a crosstalk between intracellular pathways linked to the second messenger and death receptors specifically expressed in

those cells [50, 51]. Moreover, we may speculate that different responses to cGMP may be due to cell-specific PDE5 expression levels, as well as of genes involved in the regulation of metabolic functions involving the cyclic nucleotide. Therefore, in-depth "omics" analyses should be performed to solve this unanswered question, given that *PDE5A*, *BRAF*, *KRAS*, *HRAS* and *NRAS* gene expression did not differ significantly among cell lines and thyroid cells from benign and malignant nodules (S5 Fig). Taken together, the cancer cell-specific impact of cGMP in regulating life/death signals suggests that studies should be dedicated to focus its role in cells of thyroid origin.

In thyroid tissues from biopsy samples, cGMP signaling was associated with protective mechanisms against PTC invasion [22]. These data may suggest that PDE5i could provide a new approach to treat thyroid cancer, as confirmed by other *in vitro* studies. For instance, the administration of PDE5i-loaded nanovesicles reduced the viability and proliferation of two human papillary thyroid carcinoma cell lines (TPC-1 and BCPAP) [52]. Similar results were found previously in the same cell lines, as well as in the human papillary thyroid carcinoma cell line 8505C [53]. These data were strengthened by the detection of higher PDE5 expression levels in PTC than normal thyroid tissue [53], reflecting the anti-proliferative effect of cGMP accumulation and PDE5i in thyroid cancer cells.

Although some findings may propose PDE5i as promising tools for PTC, the lack of consistent amount of data and the existence of opposite results suggest being cautious. First, the aforementioned studies evaluated the effect of PDE5i different than vardenafil, i.e. sildenafil and tadalafil, which share the same enzyme as a main target, but demonstrated different binding affinities [54, 55], and targeting of other PDEs [30, 56]. Since the PDE5 is not the unique cGMP-specific enzyme [57], these differences between PDE5i may explain tissue- and cell-specific effects. Indeed, we found no modulation of K1 and Nthy-ori 3–1 thyroid cell line viability by vardenafil, neither within μM concentrations of the compound, confirming the existence of drug- and cell-specific effects. Other explanations may rely on cGMP-independent activation of PKG, which was demonstrated using synthetic peptides [58] and occurring naturally in certain tissues [59, 60]. Interestingly, PDE5 inhibition by the drug may occur via different, cGMP-dependent and -independent intracellular mechanism. PDE5 has two highly homologous, distinct regulatory domains, GAF-A and GAF-B, which have different affinity to the inhibitor [61]. In human platelet, the sensitivity of PDE5 for sildenafil inhibition was modulated upon blockade of cGMP-binding sites of the high-affinity GAF-A domain, leading to preferential PDE5i binding to GAF-B [61] suggestive of non-canonical effects at the intracellular level.

Conclusions

We found that intracellular cGMP modulation by vardenafil does not impact the viability of benign and malignant thyroid cell models *in vitro*. While these data may suggest that the clinical use of PDE5i for erectile dysfunction could not impact the growth of thyroid cancer cells, poor but opposite results reflect the extremely cell-specific nature of the drug effect on tumors. This issue must be further investigated.

Supporting information

S1 Fig.
(TIF)

S2 Fig.
(TIF)

S3 Fig.
(TIF)

S4 Fig.
(TIF)

S5 Fig.
(TIF)

S1 File.
(DOCX)

Acknowledgments

Authors are grateful to the Italian Ministry of University and Research.

Author Contributions

Conceptualization: Manuela Simoni, Giulia Brigante, Livio Casarini.

Data curation: Sara D' Alessandro, Elia Paradiso, Clara Lazzaretti, Samantha Sperduti, Carmela Perri.

Formal analysis: Sara D' Alessandro, Elia Paradiso, Clara Lazzaretti, Samantha Sperduti, Carmela Perri, Francesco Antoniani, Sara Righi.

Investigation: Sara D' Alessandro, Elia Paradiso, Clara Lazzaretti, Samantha Sperduti, Carmela Perri, Francesco Antoniani, Sara Righi, Giulia Brigante.

Methodology: Giulia Brigante, Livio Casarini.

Project administration: Livio Casarini.

Supervision: Manuela Simoni, Giulia Brigante, Livio Casarini.

Validation: Giulia Brigante, Livio Casarini.

Visualization: Livio Casarini.

Writing – original draft: Sara D' Alessandro, Livio Casarini.

Writing – review & editing: Manuela Simoni, Giulia Brigante, Livio Casarini.

References

1. Bian K, Murad F. sGC-cGMP signaling: target for anticancer therapy. Adv Exp Med Biol. 2014; 814: 5–13. https://doi.org/10.1007/978-1-4939-1031-1_2 PMID: 25015797
2. Bian K, Ghassemi F, Sotolongo A, Siu A, Shauger L, Kots A, et al. NOS-2 signaling and cancer therapy. IUBMB Life. 2012; 64: 676–683. https://doi.org/10.1002/iub.1057 PMID: 22715033
3. Peak TC, Richman A, Gur S, Yafi FA, Hellstrom WJG. The Role of PDE5 Inhibitors and the NO/cGMP Pathway in Cancer. Sex Med Rev. 2016; 4: 74–84. https://doi.org/10.1016/j.sxmr.2015.10.004 PMID: 27872007
4. Ashour AE, Rahman AFMM, Kassem MG. Vardenafil dihydrochloride. Profiles Drug Subst Excip Relat Methodol. 2014; 39: 515–544. https://doi.org/10.1016/B978-0-12-800173-8.00009-X PMID: 24794912
5. Friebe A, Sandner P, Schmidtko A. cGMP: a unique 2nd messenger molecule—recent developments in cGMP research and development. Naunyn Schmiedebergs Arch Pharmacol. 2020; 393: 287–302. https://doi.org/10.1007/s00210-019-01779-z PMID: 31853617
6. Szakács G, Paterson JK, Ludwig JA, Booth-Genthe C, Gottesman MM. Targeting multidrug resistance in cancer. Nat Rev Drug Discov. 2006; 5: 219–234. https://doi.org/10.1038/nrd1984 PMID: 16518375

7. Lee K, Lindsey AS, Li N, Gary B, Andrews J, Keeton AB, et al. β-catenin nuclear translocation in colorectal cancer cells is suppressed by PDE10A inhibition, cGMP elevation, and activation of PKG. Oncotarget. 2016; 7: 5353–5365. https://doi.org/10.18632/ONCOTARGET.6705 PMID: 26713600

8. Wu M, Wu Y, Qian H, Tao Y, Pang J, Wang Y, et al. Type II cGMP-dependent protein kinase inhibits the migration, invasion and proliferation of several types of human cancer cells. Mol Med Rep. 2017; 16: 5729–5737. https://doi.org/10.3892/MMR.2017.7290 PMID: 28849123

9. Tinsley HN, Gary BD, Thaiparambil J, Li N, Lu W, Li Y, et al. Colon tumor cell growth-inhibitory activity of sulindac sulfide and other nonsteroidal anti-inflammatory drugs is associated with phosphodiesterase 5 inhibition. Cancer Prev Res (Phila). 2010; 3: 1303–1313. https://doi.org/10.1158/1940-6207.CAPR-10-0030 PMID: 20876730

10. Thompson WJ, Piazza GA, Li H, Liu L, Fetter J, Zhu B, et al. Exisulind induction of apoptosis involves guanosine 3',5'-cyclic monophosphate phosphodiesterase inhibition, protein kinase G activation, and attenuated beta-catenin. Cancer Res. 2000; 60: 3338–3342. PMID: 10910034

11. Fallahian F, Karami-Tehrani F, Salami S, Aghaei M. Cyclic GMP induced apoptosis via protein kinase G in oestrogen receptor-positive and -negative breast cancer cell lines. FEBS J. 2011; 278: 3360–3369. https://doi.org/10.1111/j.1742-4658.2011.08260.x PMID: 21777390

12. Windham PF, Tinsley HN. cGMP signaling as a target for the prevention and treatment of breast cancer. Semin Cancer Biol. 2015; 31: 106–110. https://doi.org/10.1016/j.semcancer.2014.06.006 PMID: 24972142

13. Wang Y, Chen Y, Wu M, Lan T, Wu Y, Li Y, et al. Type II cyclic guanosine monophosphate-dependent protein kinase inhibits Rac1 activation in gastric cancer cells. Oncol Lett. 2015; 10: 502–508. https://doi.org/10.3892/ol.2015.3173 PMID: 26171059

14. Lan T, Pang J, Wang Z, Wang Y, Qian H, Chen Y, et al. Type II cGMP-dependent protein kinase phosphorylates EGFR at threonine 669 and thereby inhibits its activation. Biochem Biophys Res Commun. 2019; 518: 14–18. https://doi.org/10.1016/j.bbrc.2019.07.126 PMID: 31395339

15. Karakhanova S, Golovastova M, Philippov PP, Werner J, Bazhin A V. Interlude of cGMP and cGMP/protein kinase G type 1 in pancreatic adenocarcinoma cells. Pancreas. 2014; 43: 784–794. https://doi.org/10.1097/MPA.0000000000000104 PMID: 24826884

16. Movsas B, Chapman JD, Greenberg RE, Hanlon AL, Horwitz EM, Pinover WH, et al. Increasing levels of hypoxia in prostate carcinoma correlate significantly with increasing clinical stage and patient age: an Eppendorf pO(2) study. Cancer. 2000; 89: 2018–2024. https://doi.org/10.1002/1097-0142(20001101)89:9<2018::aid-cncr19>3.3.co;2-p PMID: 11064360

17. Fraser M, Chan SL, Chan SSL, Fiscus RR, Tsang BK. Regulation of p53 and suppression of apoptosis by the soluble guanylyl cyclase/cGMP pathway in human ovarian cancer cells. Oncogene. 2006; 25: 2203–2212. https://doi.org/10.1038/sj.onc.1209251 PMID: 16288207

18. Thomas-Morvan C. Effect of TSH on cAMP and cGMP levels in thyroid cancers, adenomas and normal human thyroid tissue. Acta Endocrinol (Copenh). 1978; 87: 106–113. https://doi.org/10.1530/acta.0.0870106 PMID: 202130

19. Nagasaka A, Hasegawa H, Hayami S, Hidaka H, Tanaka T, Niinomi M, et al. Serum cyclic 3',5'-nucleotide phosphodiesterase in patients with various thyroid disorders. Enzyme. 1985; 33: 18–24. https://doi.org/10.1159/000469399 PMID: 2985379

20. Nagasaka A, Hidaka H. Cyclic 3',5'-nucleotide phosphodiesterase activities in the thyroid glands of patients with various disorders. J Clin Endocrinol Metab. 1980; 50: 726–733. https://doi.org/10.1210/jcem-50-4-726 PMID: 6245100

21. Van Sande J, Mockel J, Boeynaems JM, Dor P, Andry G, Dumont JE. Regulation of cyclic nucleotide and prostaglandin formation in normal human thyroid tissue and in autonomous nodules. J Clin Endocrinol Metab. 1980; 50: 776–785. https://doi.org/10.1210/jcem-50-4-776 PMID: 6245102

22. Spoto G, Esposito A, Santoleri F, Rubini C, Rutjes AWS, Fioroni M, et al. Does cyclic guanosine monophosphate induce autophagy in thyroid malignant carcinoma through down-regulation of cyclic guanosine monophosphate phosphodiesterase? J Biol Regul Homeost Agents. 2016; 30: 599–604. PMID: 27358155

23. Liang L, Luo Y, Yang X, Zhang R, Wang H, Yang H, et al. Lowered levels of microRNA-129 and potential signaling pathways in papillary thyroid carcinoma: a determination of microRNA sequencing in 507 patients and bioinformatics analysis. Int J Clin Exp Pathol. 2017; 10: 7511–7527. PMID: 31966595

24. Paradiso E, Lazzaretti C, Sperduti S, Antoniani F, Fornari G, Brigante G, et al. Sphingosine-1 phosphate induces cAMP/PKA-independent phosphorylation of the cAMP response element-binding protein (CREB) in granulosa cells. Mol Cell Endocrinol. 2021;520. https://doi.org/10.1016/j.mce.2020.111082 PMID: 33189864

25. Wyllie FS, Haughton MF, Blaydes JP, Schlumberger M, Wynford-Thomas D. Evasion of p53-mediated growth control occurs by three alternative mechanisms in transformed thyroid epithelial cells. Oncogene. 1995; 10: 49–59. PMID: 7529918

26. Powell JG, Wang X, Allard BL, Sahin M, Wang XL, Hay ID, et al. The PAX8/PPARgamma fusion oncoprotein transforms immortalized human thyrocytes through a mechanism probably involving wild-type PPARgamma inhibition. Oncogene. 2004; 23: 3634–3641. https://doi.org/10.1038/sj.onc.1207399 PMID: 15077183

27. Hamer A Den, Dierickx P, Arts R, De Vries JSPM, Brunsveld L, Merkx M. Bright Bioluminescent BRET Sensor Proteins for Measuring Intracellular Caspase Activity. ACS Sensors. 2017; 2: 729–734. https://doi.org/10.1021/acssensors.7b00239 PMID: 28670623

28. Biswas KH, Sopory S, Visweswariah SS. The GAF domain of the cGMP-binding, cGMP-specific phosphodiesterase (PDE5) is a sensor and a sink for cGMP. Biochemistry. 2008; 47: 3534–3543. https://doi.org/10.1021/bi702025w PMID: 18293931

29. Casarini L, Riccetti L, Limoncella S, Lazzaretti C, Barbagallo F, Pacifico S, et al. Probing the effect of sildenafil on progesterone and testosterone production by an intracellular FRET/BRET combined approach. Biochemistry. 2019; 58: 799–808. https://doi.org/10.1021/acs.biochem.8b01073 PMID: 30532959

30. Limoncella S, Lazzaretti C, Paradiso E, D'Alessandro S, Barbagallo F, Pacifico S, et al. Phosphodiesterase (PDE) 5 inhibitors sildenafil, tadalafil and vardenafil impact cAMP-specific PDE8 isoforms-linked second messengers and steroid production in a mouse Leydig tumor cell line. Mol Cell Endocrinol. 2022;542. https://doi.org/10.1016/J.MCE.2021.111527 PMID: 34875337

31. Casarini L, Lazzaretti C, Paradiso E, Limoncella S, Riccetti L, Sperduti S, et al. Membrane Estrogen Receptor (GPER) and Follicle-Stimulating Hormone Receptor (FSHR) Heteromeric Complexes Promote Human Ovarian Follicle Survival. iScience. 2020; 23: 101812. https://doi.org/10.1016/j.isci.2020.101812 PMID: 33299978

32. Casarini L, Riccetti L, De Pascali F, Gilioli L, Marino M, Vecchi E, et al. Estrogen modulates specific life and death signals induced by LH and hCG in human primary granulosa cells in vitro. Int J Mol Sci. 2017;18. https://doi.org/10.3390/ijms18050926 PMID: 28452938

33. Mosmann T. Rapid colorimetric assay for cellular growth and survival: application to proliferation and cytotoxicity assays. J Immunol Methods. 1983; 65: 55–63. https://doi.org/10.1016/0022-1759(83)90303-4 PMID: 6606682

34. Kaneko Y, Tsukamoto A. Thapsigargin-induced persistent intracellular calcium pool depletion and apoptosis in human hepatoma cells. Cancer Lett. 1994; 79: 147–155. https://doi.org/10.1016/0304-3835(94)90253-4 PMID: 8019972

35. B T, A T W, S R D, Isaacs JT. Thapsigargin induces a calmodulin/calcineurin-dependent apoptotic cascade responsible for the death of prostatic cancer cells—PubMed. In: Prostate. 2000 Jun 1; 43(4):303–17. Jun 2000. https://doi.org/10.1002/1097-0045(20000601)43:4<303::aid-pros10>3.0.co;2-v

36. Abou-taleb HA, Mustafa WW, Makram TS, Abdelaty LN, Salem H, Abdelkader H. Vardenafil Oral Dispersible Films (ODFs) with Advanced Dissolution, Palatability, and Bioavailability. Pharmaceutics. 2022;14. https://doi.org/10.3390/PHARMACEUTICS14030517 PMID: 35335893

37. Balmanno K, Cook SJ. Tumour cell survival signalling by the ERK1/2 pathway. Cell Death Differ. 2009; 16: 368–377. https://doi.org/10.1038/cdd.2008.148 PMID: 18846109

38. Dahmer MK. Caspases-2, -3, and -7 are involved in thapsigargin-induced apoptosis of SH-SY5Y neuroblastoma cells. J Neurosci Res. 2005; 80: 576–583. https://doi.org/10.1002/jnr.20471 PMID: 15825194

39. Song L, De Sarno P, Jope RS. Central role of glycogen synthase kinase-3beta in endoplasmic reticulum stress-induced caspase-3 activation. J Biol Chem. 2002; 277: 44701–44708. https://doi.org/10.1074/jbc.M206047200 PMID: 12228224

40. Tsai WK, Jiann BP. Data on the utilization of treatment modalities for ED in Taiwan in the era of PDE5 inhibitors. Int J Impot Res. 2014; 26: 141–145. https://doi.org/10.1038/ijir.2013.53 PMID: 24451166

41. Mujoo K, Sharin VG, Martin E, Choi BK, Sloan C, Nikonoff LE, et al. Role of soluble guanylyl cyclase-cyclic GMP signaling in tumor cell proliferation. Nitric oxide Biol Chem. 2010; 22: 43–50. https://doi.org/10.1016/j.niox.2009.11.007 PMID: 19948239

42. Kastrati I, Edirisinghe PD, Wijewickrama GT, Thatcher GRJ. Estrogen-induced apoptosis of breast epithelial cells is blocked by NO/cGMP and mediated by extranuclear estrogen receptors. Endocrinology. 2010; 151: 5602–5616. https://doi.org/10.1210/en.2010-0378 PMID: 20943808

43. Ren Y, Zheng J, Yao X, Weng G, Wu L. Essential role of the cGMP/PKG signaling pathway in regulating the proliferation and survival of human renal carcinoma cells. Int J Mol Med. 2014; 34: 1430–1438. https://doi.org/10.3892/ijmm.2014.1925 PMID: 25244411

44. Tuttle TR, Mierzwa ML, Wells SI, Fox SR, Ben-Jonathan N. The cyclic GMP/protein kinase G pathway as a therapeutic target in head and neck squamous cell carcinoma. Cancer Lett. 2016; 370: 279–285. https://doi.org/10.1016/j.canlet.2015.10.024 PMID: 26551887

45. Dhayade S, Kaesler S, Sinnberg T, Dobrowinski H, Peters S, Naumann U, et al. Sildenafil Potentiates a cGMP-Dependent Pathway to Promote Melanoma Growth. Cell Rep. 2016; 14: 2599–2610. https://doi.org/10.1016/j.celrep.2016.02.028 PMID: 26971999

46. Li WQ, Qureshi AA, Robinson KC, Han J. Sildenafil use and increased risk of incident melanoma in US men: a prospective cohort study. JAMA Intern Med. 2014; 174: 964–970. https://doi.org/10.1001/jamainternmed.2014.594 PMID: 24710960

47. Deguchi A, Thompson WJ, Weinstein IB. Activation of protein kinase G is sufficient to induce apoptosis and inhibit cell migration in colon cancer cells. Cancer Res. 2004; 64: 3966–3973. https://doi.org/10.1158/0008-5472.CAN-03-3740 PMID: 15173009

48. Huang W, Sundquist J, Sundquist K, Ji J. Phosphodiesterase-5 inhibitors use and risk for mortality and metastases among male patients with colorectal cancer. Nat Commun. 2020;11. https://doi.org/10.1038/S41467-020-17028-4 PMID: 32581298

49. Wu M, Chen Y, Jiang L, Li Y, Lan T, Wang Y, et al. Type II cGMP-dependent protein kinase inhibits epidermal growth factor-induced phosphatidylinositol-3-kinase/Akt signal transduction in gastric cancer cells. Oncol Lett. 2013; 6: 1723–1728. https://doi.org/10.3892/ol.2013.1630 PMID: 24273605

50. Kumazoe M, Sugihara K, Tsukamoto S, Huang Y, Tsurudome Y, Suzuki T, et al. 67-kDa laminin receptor increases cGMP to induce cancer-selective apoptosis. J Clin Invest. 2013; 123: 787–799. https://doi.org/10.1172/JCI64768 PMID: 23348740

51. De Nadai C, Sestili P, Cantoni O, Lièvremont JP, Sciorati C, Barsacchi R, et al. Nitric oxide inhibits tumor necrosis factor-alpha-induced apoptosis by reducing the generation of ceramide. Proc Natl Acad Sci U S A. 2000; 97: 5480–5485. https://doi.org/10.1073/pnas.070062397 PMID: 10792026

52. De Rose RF, Cristiano MC, Celano M, Maggisano V, Vero A, Lombardo GE, et al. PDE5 Inhibitors-Loaded Nanovesicles: Physico-Chemical Properties and In Vitro Antiproliferative Activity. Nanomater (Basel, Switzerland). 2016;6. https://doi.org/10.3390/nano6050092 PMID: 28335220

53. Sponziello M, Verrienti A, Rosignolo F, De Rose RF, Pecce V, Maggisano V, et al. PDE5 expression in human thyroid tumors and effects of PDE5 inhibitors on growth and migration of cancer cells. Endocrine. 2015; 50: 434–441. https://doi.org/10.1007/s12020-015-0586-x PMID: 25837309

54. Blount MA, Beasley A, Zoraghi R, Sekhar KR, Bessay EP, Francis SH, et al. Binding of tritiated sildenafil, tadalafil, or vardenafil to the phosphodiesterase-5 catalytic site displays potency, specificity, heterogeneity, and cGMP stimulation. Mol Pharmacol. 2004; 66: 144–152. https://doi.org/10.1124/mol.66.1.144 PMID: 15213306

55. Blount MA, Zoraghi R, Ke H, Bessay EP, Corbin JD, Francis SH. A 46-amino acid segment in phosphodiesterase-5 GAF-B domain provides for high vardenafil potency over sildenafil and tadalafil and is involved in phosphodiesterase-5 dimerization. Mol Pharmacol. 2006; 70: 1822–1831. https://doi.org/10.1124/mol.106.028688 PMID: 16926278

56. Mehrotra N, Gupta M, Kovar A, Meibohm B. The role of pharmacokinetics and pharmacodynamics in phosphodiesterase-5 inhibitor therapy. Int J Impot Res. 2007; 19: 253–264. https://doi.org/10.1038/sj.ijir.3901522 PMID: 16988721

57. Bork NI, Nikolaev VO. cGMP Signaling in the Cardiovascular System-The Role of Compartmentation and Its Live Cell Imaging. Int J Mol Sci. 2018;19. https://doi.org/10.3390/IJMS19030801 PMID: 29534460

58. Moon TM, Tykocki NR, Sheehe JL, Osborne BW, Tegge W, Brayden JE, et al. Synthetic Peptides as cGMP-Independent Activators of cGMP-Dependent Protein Kinase Iα. Chem Biol. 2015; 22: 1653–1661. https://doi.org/10.1016/J.CHEMBIOL.2015.11.005 PMID: 26687482

59. Neo BH, Kandhi S, Wolin MS. Roles for soluble guanylate cyclase and a thiol oxidation-elicited subunit dimerization of protein kinase G in pulmonary artery relaxation to hydrogen peroxide. Am J Physiol Heart Circ Physiol. 2010;299. https://doi.org/10.1152/ajpheart.00513.2010 PMID: 20709865

60. Neo BH, Kandhi S, Ahmad M, Wolin MS. Redox regulation of guanylate cyclase and protein kinase G in vascular responses to hypoxia. Respir Physiol Neurobiol. 2010; 174: 259–264. https://doi.org/10.1016/j.resp.2010.08.024 PMID: 20831906

61. Rybalkin SD, Rybalkina IG, Shimizu-Albergine M, Tang XB, Beavo JA. PDE5 is converted to an activated state upon cGMP binding to the GAF A domain. EMBO J. 2003; 22: 469–478. https://doi.org/10.1093/emboj/cdg051 PMID: 12554648
